# An Overview of the Biological Activity of Pyrrolo[3,4-*c*]pyridine Derivatives

**DOI:** 10.3390/ph14040354

**Published:** 2021-04-11

**Authors:** Anna Wójcicka, Aleksandra Redzicka

**Affiliations:** 1 Department of Pharmaceutical Technology, Faculty of Pharmacy, Wroclaw Medical University, Borowska 211A, 50-556 Wrocław, Poland; anna.wojcicka@umed.wroc.pl; 2 Department of Medicinal Chemistry, Faculty of Pharmacy, Wroclaw Medical University, Borowska 211, 50-556 Wrocław, Poland

**Keywords:** pyrrolo[3,4-*c*]pyridine, pyrrole heterocycles, biological activity, structure-activity relationships, analgesic activity, azaizoindoles, anticancer activity

## Abstract

Pyrrolo[3,4-*c*]pyridine is one of the six structural isomers of the bicyclic ring system containing a pyrrole moiety fused to a pyridine nucleus. The broad spectrum of pharmacological properties of pyrrolo[3,4-*c*]pyridine derivatives is the main reason for developing new compounds containing this scaffold. This review presents studies on the biological activity of pyrrolo[3,4-*c*]pyridines that have been reported in the scientific literature. Most of these derivatives have been studied as analgesic and sedative agents. Biological investigations have shown that pyrrolo[3,4-*c*]pyridines can be used to treat diseases of the nervous and immune systems. Their antidiabetic, antimycobacterial, antiviral, and antitumor activities also have been found.

## 1. Introduction

**Figure 1:**
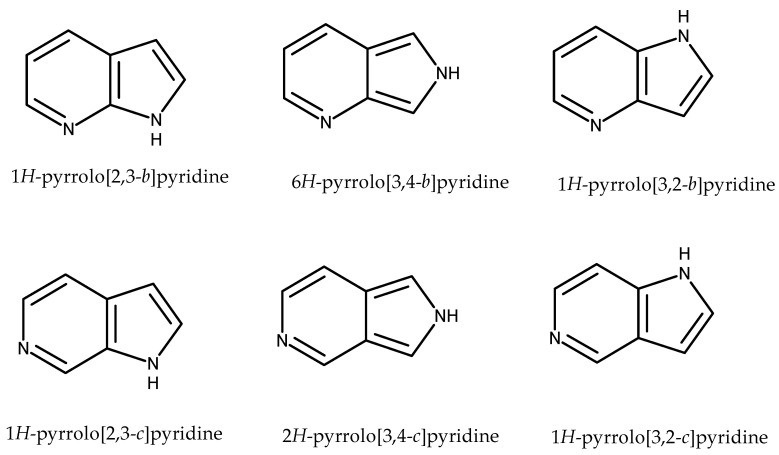
Isomeric forms of pyrrolopyridines.

Pyrrolopyridines are heterocyclic compounds containing a five-membered pyrrole ring fused to a six-membered pyridine ring. They are also called azaindoles or azaizoindoles, depending on the arrangement of nitrogen atoms in the bicyclic structure. Pyrrolopyridines occur in six isomeric forms (Figure 1).

**Figure 2:**
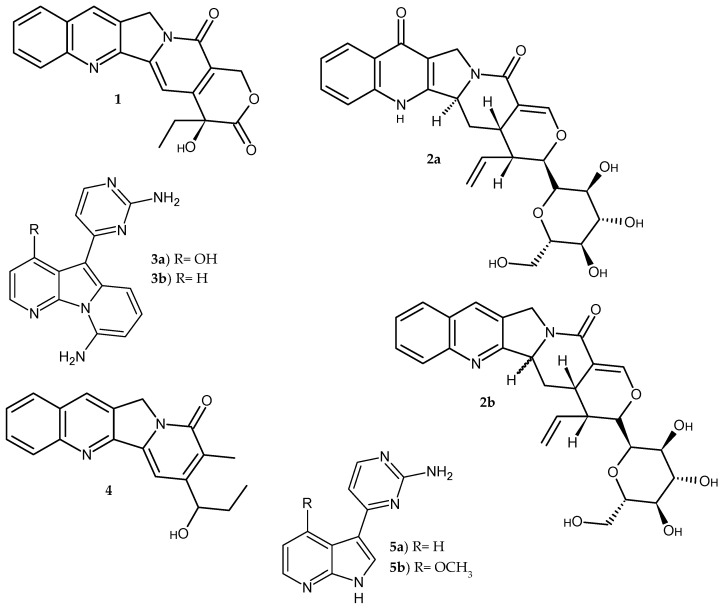
Alkaloids **1**–**5** containing pyrrolopyridine scaffold.

The pyrrolopyridine scaffold occurs in polyheterocyclic compounds of natural origin. The best known is the alkaloid camptotheca **1**, isolated from the *Camptotheca acuminata* tree. As a topoisomerase I inhibitor, it has found application in cancer treatment and is active against HIV-1 [1]. Other alkaloids containing the pyrrolopyridine system in their structure include pumiloside **2a**, deoxypumiloside **2b** isolated from *Ophiorrhiza pumila* [2], and variolin B **3a**, deoxyvariolin B **3b** isolated from an antarctic sponge *Kirkpatrickia variolosa* [3], and the antiviral alkaloid mappicin **4**, which was isolated from *Mappia foetida* [4], and anticancer meriolin derivatives **5a–b** [3] (Figure 2).

Due to their structure containing two important pharmacophores, i.e., pyrrole and pyridine, pyrrolopyridines have been the subject of many pharmacological studies over the last hundred years. Pyrrole derivatives show a wide range of pharmacological activity. Pyrroles could build the structure of porphyrin rings. Many of the drugs contain a pyrrole ring, for example, nootropic (Piracetam), anti-inflammatory (Indometacin), statins (Fluvastatin), neuroleptics (Molindone), drugs used to treat insomnia (Zopiclone), migraine (Sumatriptan), Parkinson disease (Procyclidine), and erection disorders (Tadalafil). Compounds containing a pyrrole ring found in essential oils are used as anticancer agents (5-iodotubercidin) [5] and antibacterial agents (Cefepim, Doripenem, Meropenem) [6]. Fostemsavir has recently been approved for HIV treatment. Drugs containing a pyrrolopyridine scaffold such as Vemurafenib or Pexidartinib are used in anticancer therapy [3]. Pyrrolo[3,2-*c*]pyridine derivatives show inhibitory effect against FMS kinase and are promising candidates for anticancer and antiarthritic drug development [7].

This review presents biologically active derivatives of the pyrrolo[3,4-*c*]pyridine isomer. Pyrrolo[3,4-*c*]pyridine derivatives have not been introduced into medicine so far, but as it results from studies presented in the literature, many of them show biological activity.

## 2. Biological Activity of Pyrrolo[3,4-*c*]pyridine Derivatives

### 2.1. Antidiabetic Activity of Pyrrolo[3,4-c]pyridine Derivatives

**Figure 3:**
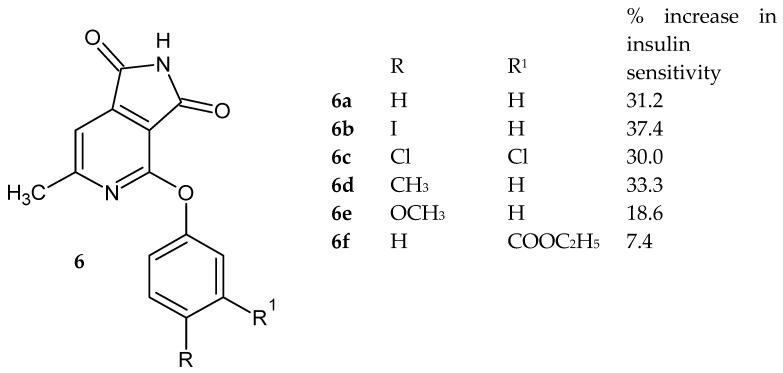
6-Methyl-pyrrolo[3,4-*c*]pyridine-1,3(*2H*)-dione derivatives **6a**–**f** increased the insulin sensitivity.

Knutsen et al. [8] described 4-substituted 6-methyl-pyrrolo[3,4-*c*]pyridine-1,3(*2H*)-dione derivatives **6** (Figure 3) that effectively reduce blood glucose levels without affecting the concentration of circulating insulin. The present compounds reduce blood glucose levels by stimulating glucose uptake into muscle and fat cells.

The ability of pyrrolo[3,4-*c*]pyridine-1,3(*2H*)-dione derivatives **6** to stimulate the incorporation of glucose into lipids was tested. The maximum increase in insulin sensitivity achieved in the dose range of 0.3–100 µM of the tested compounds, normalized to the full insulin dose-response (100%), was determined. The phenoxy substituent in the 4-position turned out to significantly influence the activity of the derivatives. While 4-phenoxy-6-methyl-pyrrolo[3,4-*c*]pyridine-1,3(*2H*)-dione derivatives increased the insulin sensitivity of mouse adipocytes by 7.4–37.4% (Figure 3). The substituent in the para position of the phenyl ring increased the activity. Compounds with 4-phenoxy-(**6a**), 4-(4-iodophenoxy)-(**6b**), 4-(3,4-dichlorophenoxy)-(**6c**) and 4-(4-methylphenoxy)-(**6d**) derivatives increased the insulin sensitivity by more than 30% [8].

Due to the efficacy of the present compounds **6** to reduce the blood glucose, they may find application in the prevention and treatment of disorders involving elevated plasma blood glucose, such as hyperglycemia and ailments in which such a reduction of blood glucose is beneficial: type 1 diabetes, diabetes as a consequence of obesity, diabetic dyslipidemia, hypertriglyceridemia, insulin resistance, impaired glucose tolerance, hyperlipidemia, cardiovascular diseases, and hypertension.

Aldose reductase (AR) is an enzyme present in the lens and brain that eliminates excess glucose by catalyzing glucose reduction to sorbitol. The accumulation of sorbitol may cause neuropathy in the peripheral nerves and cataracts in the lens. AR inhibitors reduce secondary complications caused by diabetes mellitus, especially in tissues where glucose uptake is not insulin-dependent.

**Figure 4:**
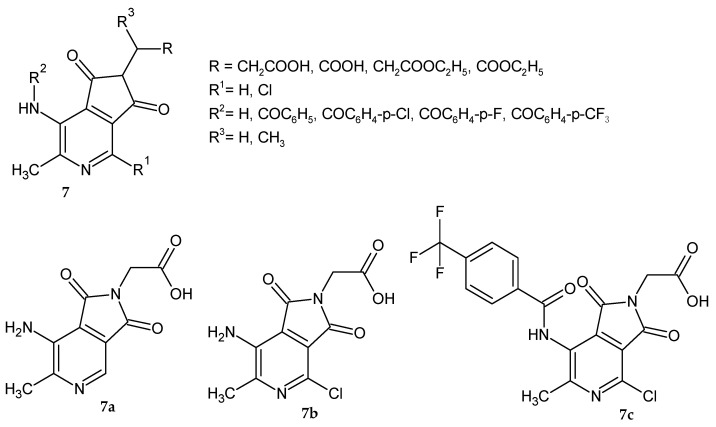
6-Methyl-pyrrolo[3,4-*c*]pyridine-1,3-dione derivatives **7** as aldose reductase inhibitors and the most active derivatives **7a**–**c**.

Da Settimo et al. [9] synthesized a series of 6-methylpyrrolo[3,4-*c*]pyridine-1,3-dione alkanoic acid derivatives **7** with potential AR inhibitory activity (Figure 4). The obtained compounds were evaluated for their ability to inhibit AR in vitro in the extract of rat lenses. The biological data showed that the acidic derivatives proved to be new AR inhibitors. The presence of the carboxylic group at an adequate distance from the pyrrolopyridine scaffold is important for their activity. Compounds with a small chain length of carboxylic acid proved to be much more potent inhibitors than propionic or iso-propionic acid derivatives. Its corresponding ethyl ester analogs were inactive even at a higher concentration range. Among amide analogs most potent was 4-trifluoromethyl-benzylamide derivative **7c**, but no more active than **7a**. The inhibition potency of compounds **7a**–**c** was similar to the reference Alrestatin and Sorbinil at concentrations: 10^−5^ M (70–90%) and 10^−6^ M (33–43%). IC_50_ (the concentration at which a substance exerts half of its maximal inhibitory effect) values for compounds **7a**–**c** were 1.4 µM, 2.5 µM, and 1.7 µM, respectively. The most active derivatives **7a**–**b** were evaluated as inhibitors of glutathione lens depletion in galactosemic rats. Glutathione depletion in the lens occurs rapidly after induction of galactosemia in rats (following the galactose diet). None of the tested compounds were active in maintaining glutathione levels in the rats’ lenses, which may be due to problems with metabolism or ocular bioavailability.

GPR119 is a cannabinoid receptor expressed predominantly in the incretin-releasing intestinal cells and pancreatic islet β-cells. GPR119 regulates the secretion of incretin and insulin. Therefore, new drugs acting on the GPR119 could find application in treating obesity and type 2 diabetes.

**Figure 5:**
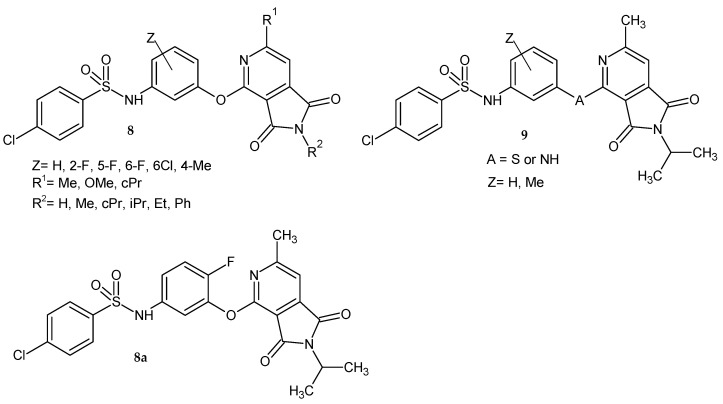
Pyrrolo[3,4-*c*]pyridine-1,3-dione derivatives **8**–**9** with 4-chloro-N-phenyl-benzenesulfonamide substituent as GPR119 agonists and the most active compound **8a**.

Yu et al. [10] synthesized a series of N-[3-(1,3-dioxo-2,3-dihydro-*1H*-pyrrolo[3,4-*c*]pyridin-4-yloxy)phenyl]benzenesulfonamide derivatives **8** as novel GPR119 agonists. They replaced the -O- linker with the -S- or -NH-, as exemplified in **9**, but both modifications did not bring any benefit, only an efficacy reduction of about 20% (Figure 5).

Scientists investigated the effect of pyrrolo[3,4-*c*]pyridine ring substituents on potency [10]. The methyl group for R^1^ was selected for its potential to improve metabolic stability. Biological studies have shown that the potency of derivatives 8 also depends on the R^2^ substituent. Activity increased for the ethyl and propyl chains as R^2^ compared to the methyl substituent or lack thereof, but large substituents (e.g., phenyl, cyclopentyl) lead to a significant loss of potency. Pharmacokinetic studies showed different metabolic decomposition of the tested compounds in both rat liver microsomes (RLM) depending on the substitution of the middle phenyl ring. The most active 6-F-phenyl derivative 8a exhibited an EC_50_ of 0.016 µM in the human GPR119 cAMP assay and good RLM stability with clearance intrinsic (CL- the liver ability to remove (metabolize) a drug without the restrictions placed on it being delivered to the liver cell by blood flow or protein binding) = 84 µL/min/mg. Compound **8a** was also evaluated during in vivo experiments and exhibited 0.073 µM in the cynomolgus monkey GPR119 cAMP assay with 100% efficacy, and in human liver microsomes, the CL was 29 µL/min/mg. Derivative **8a** showed good bioavailability in rats (95%) and 29% in cynomolgus monkeys following a 2.0 mg/kg PO dose. It did not bind to BSEP (bile salt export pump), did not show hPXR (the human pregnane X receptor) activation and cytochrome P450 inhibition. Although the pharmacological properties were excellent, compound **8a** showed high plasma protein binding, which precluded the assessment of its antidiabetic efficacy in vivo [10].

### 2.2. Antimicrobial Activity of Pyrrolo[3,4-c]pyridines

The growing problem of bacterial resistance prompts the search for new antibacterial compounds. Pyrrolo[3,2-b]pyridine derivatives show activity against resistant strains of *E. coli* [11], which stimulates the search for new pyrrolopyridine derivatives with antimicrobial activity.

#### 2.2.1. Antiviral Activity of Pyrrolo[3,4-*c*]pyridine Derivatives

HIV-1 integrase (IN) catalyzes the incorporation of HIV-1 cDNA into host DNA through a process involving two steps: processing of the HIV DNA 3′ ends (3′-P) and strand transfer (ST). Due to the emergence of raltegravir (an IN inhibitor) resistance, it is imperative to develop second-generation inhibitors that will remain effective against clinically significant IN mutants.

**Figure 6:**
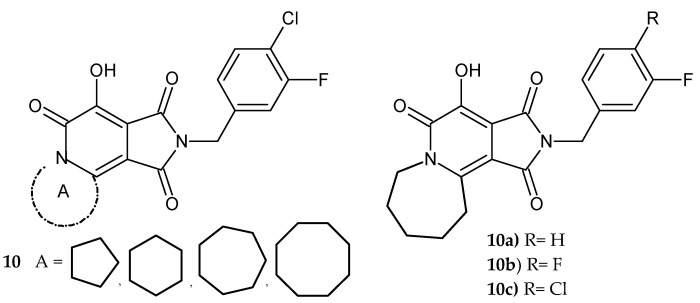
Tricyclic compounds containing pyrrolo[3,4-*c*]pyridine scaffold **10** as HIV-1 integrase inhibitors and the most active compounds **10a**–**c**.

Researchers under Zhao [12] developed a series of new IN inhibitors containing the pyrrolo[3,4-*c*]pyridine scaffold. Tricyclic compounds **10** (N-benzyl-7-hydroxy-pyrrolo[3,4-*c*]pyridine-1,3,6-trione nucleus condensed with a ring ranging in size from five to eight members) (Figure 6) demonstrated low micromolar inhibitory potency in IN assays in vitro (inhibition potency slightly increased with ring size), good selectivity (for the ST step relative to the 3′-P step), and reduced cytotoxicity (Figure 6). These analogs **10a**–**c** were less sensitive than raltegravir to resistance induced by mutations in integrase.

**Figure 7:**
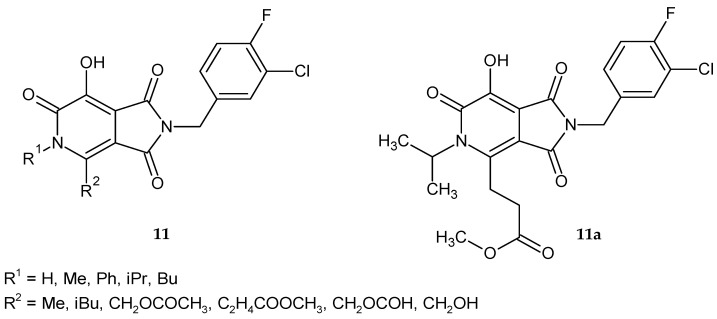
2-(3-Chloro-4-fluorobenzyl)-7-hydroxy-1*H*-pyrrolo[3,4-*c*]pyridine-1,3,6(*2H,5H*)-trione derivatives **11** and the most potent compound **11a**.

Zhao et al. [13] also synthesized bicyclic compounds: 2-(3-chloro-4-fluorobenzyl)-7-hydroxy-1*H*-pyrrolo[3,4-*c*]pyridine-1,3,6(2*H*,5*H*)-trione derivatives **11** (Figure 7) and assessed them in biochemical IN tests that measure the inhibitory power. The potency of new compounds was similar (IC_50_ values for the ST reactions in the low micromolar range: 6–22 μM). Methyl 3-(2-(3-chloro-4-fluorobenzyl)-7-hydroxy-5-isopropyl-1,3,6-trioxo-2,3,5,6-tetrahydro-1*H*-pyrrolo[3,4-*c*]pyridin-4-yl)propanoate **11a** retained most of its inhibitory potency against the three raltegravir-resistance mutant IN enzymes. In antiviral assays, it was approximately 200-, 20-, and 10-fold less affected than raltegravir concerning G140S/Q148H, Y143R, and N155H mutations, respectively [13].

The biological data suggest that the pyrrolo[3,4-*c*]pyridine-1,3,6-trione scaffold can be used to create compounds insensitive to mutations of HIV-1 integrase that have a real potential in AIDS treatment.

**Figure 8:**
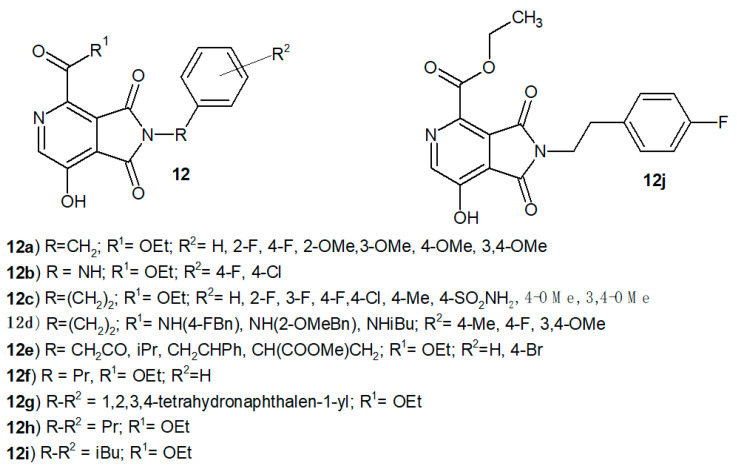
7-Hydroxy-1,3-dioxo-2,3-dihydro-1*H*-pyrrolo[3,4-*c*]pyridine-4-carboxylate derivatives **12** and the most active compound **12j**.

Researchers led by Liu [14] synthesized a series of 7-hydroxy-1,3-dioxo-2,3-dihydro-1*H*-pyrrolo[3,4-*c*]pyridine-4-carboxylates **12a**–**i** (Figure 8) and evaluated their anti-HIV-1 activity. Most of the obtained compounds showed moderate activity in inhibiting HIV-1 replication. It turned out that the ester substituent at position 4 has a significant influence on the activity of derivatives. The distance between the pyrrolopyridine scaffold and the phenyl ring proved to be equally important. Derivatives in which R = ethyl, R^1^= OEt and R^2^= Ph, 4-FPh, 4-MePh or indol-3-yl exhibited potent activity (EC_50_ <10 µM). The most active ethyl 2-(4-fluorophenethyl)-7-hydroxy-1,3-dioxo-2,3-dihydro-*1H*-pyrrolo[3,4-*c*]pyridine-4-carboxylate **12j** showed significant anti-HIV-1 activity (EC_50_ = 1.65 µM) and in vitro therapeutic index TI = 7.98 [14].

**Figure 9:**
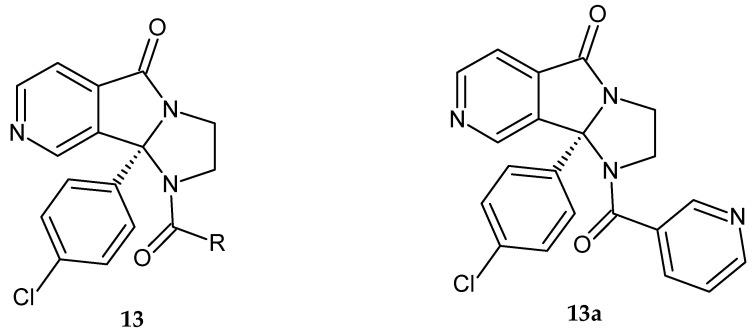
Imidazo[1’,2’:1,2]pyrrolo[3,4-*c*]pyridin-5-one derivatives **13** with anti-RSV activity and the candidate for preclinical studies **13a**.

Respiratory syncytial virus (RSV), also called *human orthopneumovirus,* causes infections of the respiratory tract. Bond et al. [15] synthesized 9b-(4-chlorophenyl)-1,2,3,9b-tetrahydro-5*H*-imidazo[1’,2’:1,2]pyrrolo[3,4-*c*]pyridin-5-one derivatives **13** (Figure 9). The obtained compounds were evaluated for their activity against RSV in a cytopathic effect assay. The most potent derivatives were also tested for the presence of alpha-1-acid glycoprotein to evaluate the binding effect to a human serum protein. The effect of the substituent R was also investigated. Furan, pyrazole, pyrrole, and pyridine derivatives were most active against RSV and showed good protein binding properties. The S-enantiomer of the tested compounds turned out to be active. Derivatives combining excellent anti-RSV activity with good protein binding properties were tested for RSV cross-strain activity, solubility, lipophilicity, and pharmacokinetic properties in rats. All compounds exhibited good activity against the three RSV strains, acceptable solubility, and low lipophilicity. The pyridine derivative **13a** showed excellent bioavailability and a terrific pharmacokinetic (PK) profile at high exposure. Virological studies showed that derivative **13a** is a promising direct and selective RSV fusion inhibitor and has been selected for preclinical studies as a candidate for RSV infection treatment [15].

#### 2.2.2. Antimycobacterial Activity of Pyrrolo[3,4-*c*]pyridine Derivatives

*Mycobacterium tuberculosis* (Mtb) is a species of pathogenic bacteria with a waxy coating on the surface of cells, mainly due to mycolic acid presence. Isoniazid, one of the most effective anti-tuberculosis drugs, affects the synthesis of mycolic acids. It inhibits enoyl-acyl carrier protein reductase (InhA), an enzyme from *Mycobacterium tuberculosis*, which is one of the key enzymes involved in the type II fatty acid biosynthesis pathway (FAS II) system. Ethionamide also exerts an antimycobacterial activity by inhibiting InhA.

**Figure 10:**
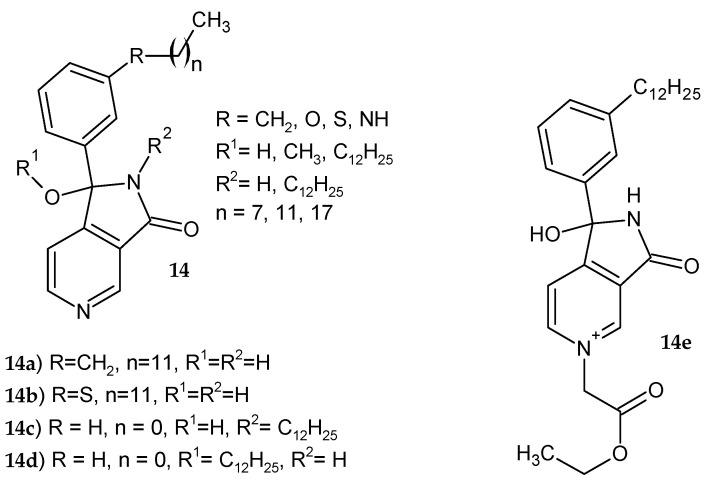
1-Phenyl-pyrrolo[3,4-*c*]pyridine-3-one derivatives **14a**–**e** as InhA enzyme inhibitors.

Deraeve et al. [16] synthesized pyrrolo[3,4-*c*]pyridine-3-one derivatives **14** as new inhibitors of the InhA enzyme (Figure 10). The obtained compounds were screened in vitro at 100 and 30 µM, and the InhA inhibition percentage, MIC (minimum inhibitory concentration), and IC_50_ were determined against *M. tuberculosis*. The substitution of the phenyl ring was investigated. Unsubstituted or meta derivatives were most active. The chain substitution at the ortho position abolished this activity. Replacing the methyl or sulfur linker with oxygen or an NH group resulted in a decreased activity. On the other hand, dodecane derivative **14a** is not an effective inhibitor of InhA (4% at 100 µM dose), proving that the presence of the electronegative atom is important for binding affinity. The most active derivatives **14a-d** exhibited MIC <25 µM and IC_50_ values in the range of 4.5–9.5 µM [11]. Derivative **14e** obtained by Delaine et al. [17] inhibited 91% of InhA activity at 100 µM and 40% at 20 µM. Compound **14e** exhibited higher growth inhibition of *M. smegmatis* than isoniazid but was less active against *M. tuberculosis*.

**Figure 11:**
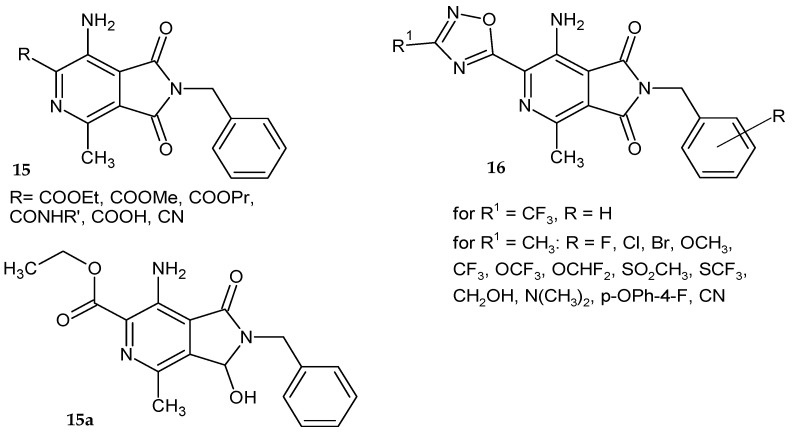
7-Amino-2-benzyl-4-methyl-pyrrolo[3,4-*c*]pyridine-1,3-dione derivatives **15**–**16** with an antimycobacterial activity and the most active compound **15a**.

Westhuyzen et al. [18,19] synthesized a series of 7-amino-2-benzyl-4-methyl-1,3-dioxo-2,3-dihydro-1*H*-pyrrolo[3,4-*c*]pyridine derivatives **15**–**16** (Figure 11) and assessed their antimycobacterial activity. Nitriles and amides **15** were not active with MIC_90_ (the lowest concentration of the drug inhibiting the growth of over 90% of the bacterial population) above 160 µM, while the esters exhibited good activity in vitro (MIC_90_ <0.15 µM). The moderately active carboxylic acid (MIC_90_ = 3.13 µM) showed significantly improved solubility and microsomal metabolic stability. Compounds 16 with the 1,2,4-oxadiazole moiety instead of the ester group showed better in vitro activity against *Mtb* and metabolic stability, but the 1,3,4-oxadiazole derivative exhibited a lower activity. The 3-hydroxy analog **15a** exhibited good activity in vitro and improved solubility. Removal of the methyl group from the pyridine ring resulted in a significant loss of activity. All tested compounds were not toxic to VERO cells. The most active, stable and highly soluble 7-amino-2-(3-chlorobenzyl)-4-methyl-6-(3-methyl-1,2,4-oxadiazol-5-yl)-1*H*-pyrrolo[3,4-*c*]pyridine-1,3-dione **16** was evaluated using an in vivo pharmacokinetic study. Mouse PK studies showed low plasma exposure and high clearance compared to good metabolic stability in liver microsomes.

#### 2.2.3. Antibacterial and Antifungal Activity of Pyrrolo[3,4-*c*]pyridine Derivatives

**Figure 12:**
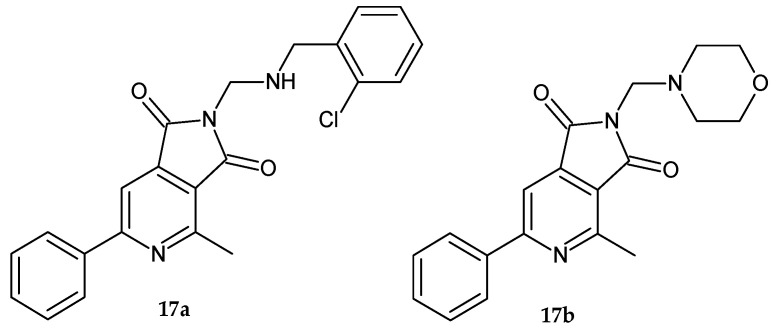
Mannich bases **17a–b** displayed activity against *S. aureus* and *C. albicans.*

Mannich bases **17a–b** (Figure 12) of 4-methyl-6-phenylpyrrolo[3,4-*c*]pyridine-1,3-dione obtained by Wojcicka et al. [20] exhibited moderate activity against *Staphylococcus aureus.* Compound **17a** also reduced the growth of *Candida albicans* in a statistically significant manner.

### 2.3. Anticancer Activity of Pyrrolo[3,4-c]pyridine Derivatives

**Figure 13:**
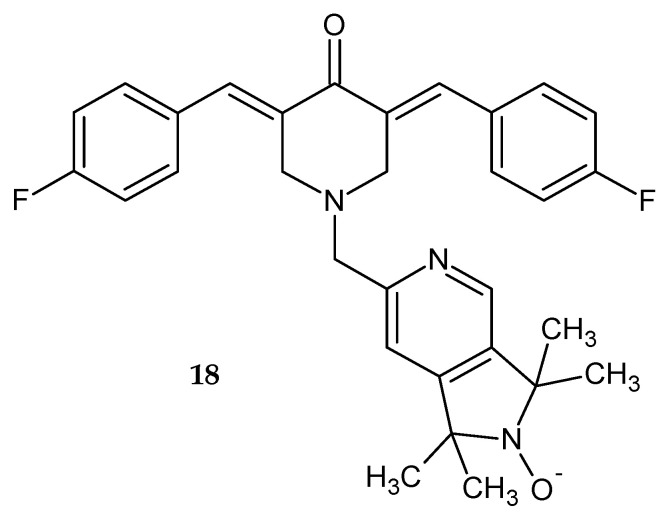
N-oxide 3,5-bis(4-fluorobenzylidene)-1-(1,1,3,3-tetramethyl-1,2-dihydropyrrolo[3,4-*c*]pyridin-6-yl)piperidin-4-on **18**.

In a study by Kalai et al. [21], 3,5-bis(4-fluorobenzylidene)-1-(1,1,3,3-tetramethyl-1,2-dihydropyrrolo[3,4-*c*]pyridin-6-yl)piperidin-4-on N-oxide **18** was synthesized (Figure 13). The antitumor activity of the obtained compound was assessed by measuring its cytotoxicity against ovarian and breast cancer cell lines and noncancerous cardiac cell lines. Tested compound **18** exhibited moderate cytotoxicity against ovarian cancer cells and limited toxicity toward breast cancer and healthy cardiac cell lines [21].

Nicotinamide phosphoribosyltransferase (NAMPT) catalyzes the condensation of nicotinamide (NAM) with 5-phosphoribosyl-1-pyrophosphate to nicotinamide mononucleotide (NMN), which is the first step in NAD+ biosynthesis. Blocking NAMPT activity may impair cell growth. Thus, NAMPT inhibition is one of the anti-cancer therapy strategies.

**Figure 14:**
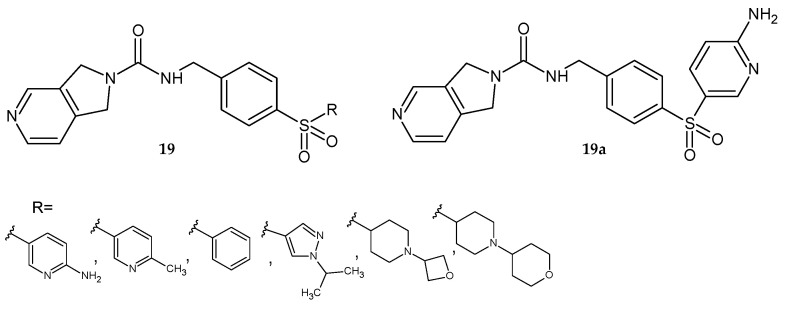
4-Sulfonylobenzyl- derivatives of pyrrolo[3,4-*c*]pyridine-2-carboxamide **19** as potent inhibitors of NAMPT and the most active compound **19a**.

Dragovich et al. [22] synthesized 4-sulfonylobenzyl- derivatives of pyrrolo[3,4-*c*]pyridine-2-carboxamide **19** (Figure 14) and evaluated them as potent NAMPT inhibitors. Derivatives that showed strong activity against NAMPT, good in vitro stability against human liver microsomes, and sufficient water solubility were referred for further studies. Many of these compounds exhibited minimal inhibition of the cytochrome P450 3A4 isoform and several CYP isoforms (2D6, 1A2, 2C19), but were strong CYP2C9 inhibitors. The binding of these derivatives to mouse plasma protein was typically moderate (approximately 90% bound). Molecule **19a** also showed antiproliferative effects against several human tumor cell lines. Based on that data, compound **19a** was accepted for in vivo mouse PK and xenograft efficacy studies. The **19a** derivative showed satisfactory PK properties in mice and was effective in the PC-3 mouse xenograft model. Derivative **19a** exhibited good bioavailability and plasma exposure after oral administration to female NCR mice [22].

**Figure 15:**
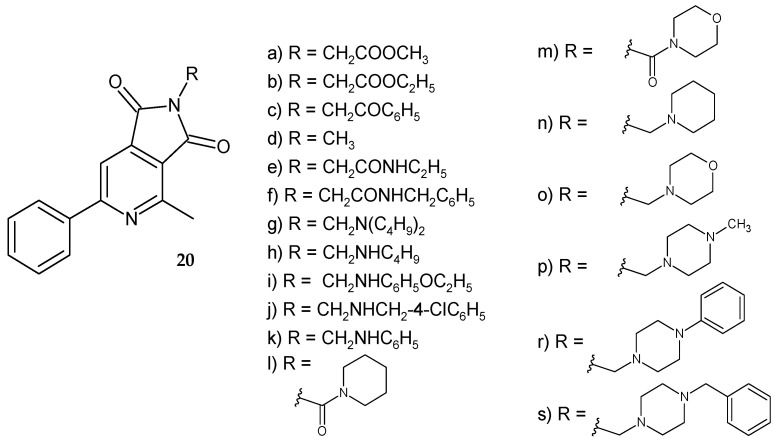
*N*-alkil-4-methyl-6-phenyl-1*H*-pyrrolo[3,4-*c*]pyridine-1,3-dione derivatives **20a**–**s**.

Wojcicka et al. [20] synthesized a series of 4-methyl-6-phenylpyrrolo[3,4-*c*]pyridine-1,3-dione derivatives by modifying of the substituent at position 2 of the pyrrolo[3,4 *c*]pyridine scaffold (Figure 15). *N*-alkil-4-methyl-6-phenyl-1*H*-pyrrolo[3,4-*c*]pyridine-1,3-diones **20a**–**s** were obtained and screened for their antitumor activity in vitro. Mannich bases **20g**–**s** with an IC_50_ value in the range of 19–29 µg/mL were the most active.

Phosphoinositide 3-kinases (PI3Ks) are a family of intracellular lipid signaling enzymes that catalyze the phosphorylation of the hydroxyl group in the 3-position of the phosphatidylinositol ring. PI3Ks can control many vital cellular processes such as growth, proliferation, and survival. Therefore, the inhibition of PI3Ks can be used in anticancer therapy.

**Figure 16:**
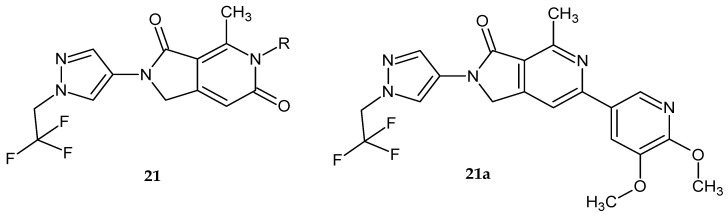
4-Methyl-2-[1-(2,2,2,-trifluoroethyl)-1*H*-pyrazol-4-yl]-1*H*-pyrrolo[3,4-*c*]pyridin-3-one derivatives **21** and compound **21a** as the potent inhibitor of PI3Ks.

Collier et al. [23] synthesized 4-methyl-2-[1-(2,2,2,-trifluoroethyl)-1*H*-pyrazol-4-yl]-1*H*-pyrrolo[3,4-*c*]pyridine-3,6-dione derivatives **21** (Figure 16). The substitution of the carbonyl group in the 6-position with a 5,6-dimethoxypyridin-3-yl ring led to derivative **21a**, which showed high PI3Kγ affinity and inhibited the monocyte chemoattractant protein-1 MCP-1 induced chemotaxis of THP-1 cells (IC_50_ = 270 nM).

Spleen tyrosine kinase (SYK) is a non-receptor cytoplasmic kinase that is a key mediator for various inflammatory cells. Therefore its inhibition is an essential therapeutic target in both neoplastic and inflammatory diseases. FLT3 (fms like tyrosine kinase 3) is a cytokine receptor expressed on the surface of many hematopoietic progenitor cells.

**Figure 17:**
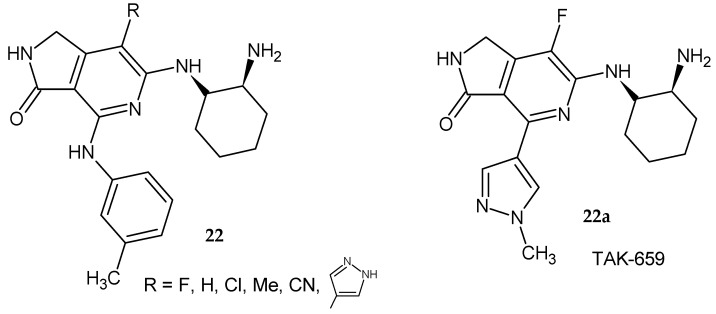
6-[(2-Aminocyclohexyl)amino]-1,2-dihydro-3*H*-pyrrolo[3,4-*c*]pyridin-3-one derivatives **22** as SYK inhibitors and compound **22a** used in clinical trials.

Lam et al. [24] prepared 6-[(2-aminocyclohexyl)amino]-1,2-dihydro-3*H*-pyrrolo[3,4-*c*]pyridin-3-one derivatives **22** as SYK inhibitors (Figure 17). The 4-(3-methylanilino)-substituted compounds exhibited good enzymatic and cellular potency. It turned out that the 7-fluoro substitution had a significant effect on the activity. Replacing methylaniline with a methylpyrazole ring increased the activity of the 4-fluoro derivative. The methylpyrazole derivative **22a** showed the best in vitro profile and was selective for kinases (it was potent toward both SYK and FLT3). Compound **22a** showed inhibition toward an SYK-dependent and FLT3-dependent cell line in a cell proliferation assay. It demonstrated strong tumor growth inhibition (TGI) after 20 days of treatment. This derivative blocked anti-IgD stimulated expression of CD86 in murine peripheral B cells in vivo. Compound **22a** named TAK-659 has been used in clinical trials to treat advanced solid tumors and lymphoma malignancies [24].

### 2.4. Anti-Inflammatory Activity of Pyrrolo[3,4-c]pyridine Derivatives

Matrix metalloproteinases (MMPs) are zinc-dependent proteolytic enzymes involved in the degradation and regeneration of the extracellular matrix. High levels of MMPs may cause a variety of pathologies, including metastatic cancer and arthritis, and therefore inhibition of these enzymes is considered an important therapeutic target.

**Figure 18:**
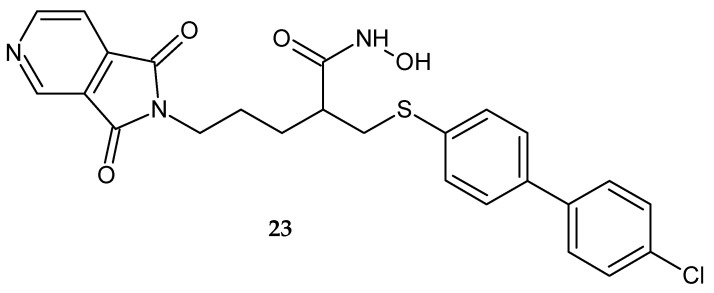
5-(1,3-Dioxo-1,3-dihydro-2*H*-pyrrolo[3,4-*c*]pyridin-2-yl)-2-{[(4’-chloro[1,1’-biphenyl]-4-yl)sulfanyl]methyl}-*N*-hydroxypentanamide **23**.

Chollet et al. [25] synthesized 5-(1,3-dioxo-1,3-dihydro-2*H*-pyrrolo[3,4-*c*]pyridin-2-yl)-2-{[(4’-chloro[1,1’-biphenyl]-4-yl)sulfanyl]methyl}-*N*-hydroxypentanamide **23** (Figure 18) as an inhibitor of MMPs with IC_50_ = 3 nM for MMP-2, IC_50_ = 12 nM for MMP-9 and IC_50_ = 84 nM for MMP-13.

**Figure 19:**
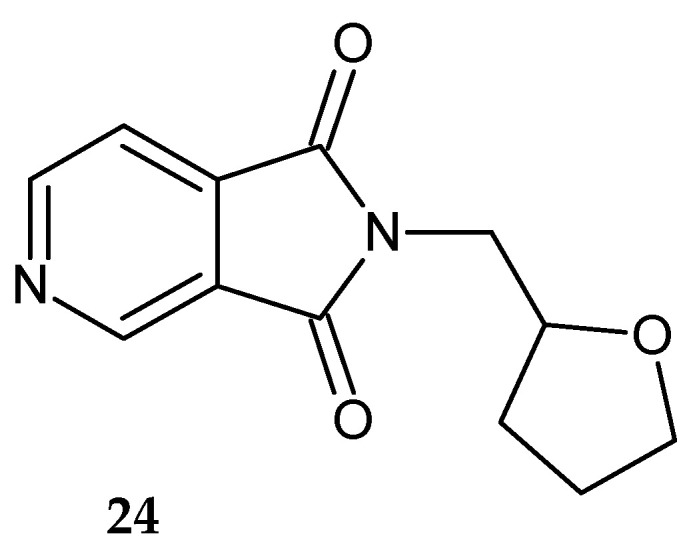
2-((Tetrahydrofuran-2-yl)methyl)-2*H*-pyrrolo[3,4-*c*]pyridine-1,3-dione **24**.

Sondhi et al. [26] synthesized 2-((tetrahydrofuran-2-yl)methyl)-2*H*-pyrrolo[3,4-*c*]pyridine-1,3-dione **24** (Figure 19) and evaluated it for anti-inflammatory activity. Compound **24** showed 26% activity at a dose of 50 mg/kg per os.

### 2.5. Sedative Activity of Pyrrolo[3,4-c]pyridine Derivatives

While conducting studies on pyrrolo[3,4-*c*]pyridines to obtain new anxiolytic derivatives of the buspirone type, Śladowska’s team noticed that these compounds are often not active in this regard. Instead, they show other pharmacological effects, in particular, sedative [27] and analgesic activity [27,28,29]. The analgesic activity of the new pyrrolo[3,4-*c*]pyridines is described in the following section.

**Figure 20:**
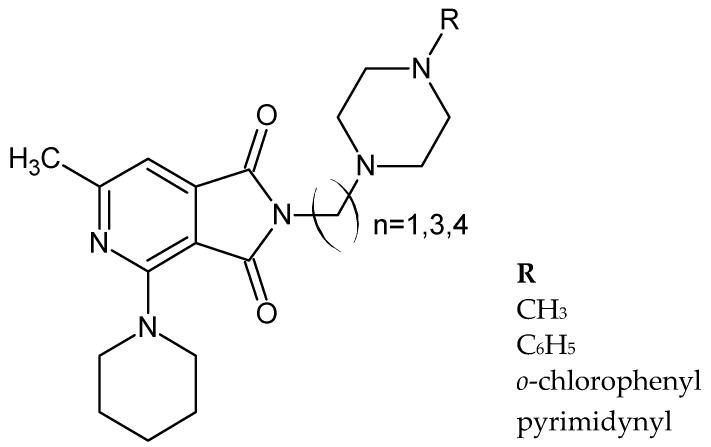
General structure pyrrolo[3,4-*c*]pyridines [27].

Taking into account the results of previous work [28,29], in 1994, the above researchers obtained a series of nine new N-substituted piperazinalkyl derivatives of 6-methyl-2-(1-piperidine)-pyridine-1,3-dione (Figure 20) [27].

Several pharmacological studies were performed with regard to the newly obtained structures, including testing for:

- acute toxicity in mice;
- spontaneous locomotor activity in mice;
- pantetrazol-induced seizures in mice;
- anxiolytic properties in a four-plate test in mice;
- amphetamine-induced motor hyperactivity in mice;
- pain reactivity in a writhing syndrome test in mice.

**Figure 21:**
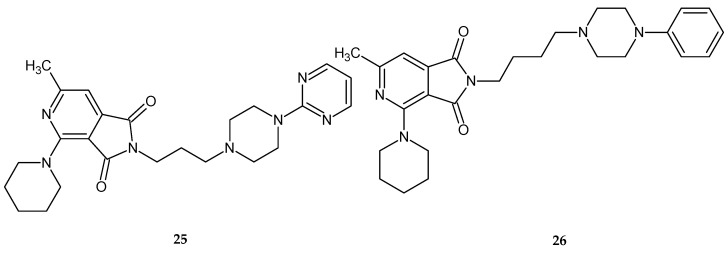
Derivatives of pyrrolo[3,4-*c*]pyridines **25** and **26** with significantly sedative activity [27].

The test results showed that out of the tested series of compounds, derivatives **25** and **26** (Figure 21) significantly decreased locomotor activity during a one-hour observation.

Compound 25 showed this effect at doses of 1/10 and 1/20 LD_50_, 26 at doses of 1/10, 1/20, 1/40, and even 1/80 of LD_50_ [27]. In the other tests mentioned above, the activity of compounds was not as promising. None of the compounds tested at doses of 1/10 LD_50_ showed antinociceptive activity in the writhing test [27].

**Figure 22:**
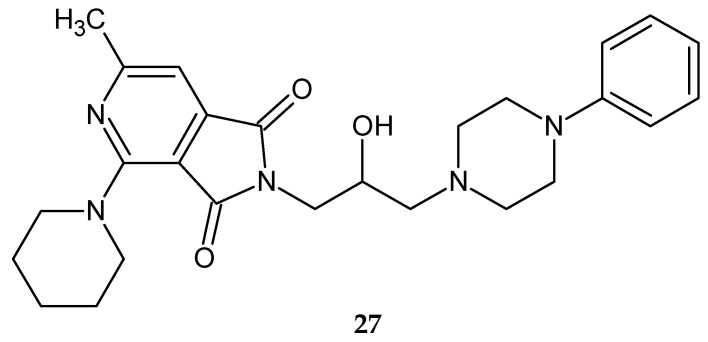
2-[2-hydroxy-3-(4-phenyl-1-piperazinyl)propyl]-4-piperidino-6-methyl-1*H*-pyrrolo[3,4-*c*]pyridine-1,3(2*H*)-dione **27**.

A year later, the same scientists obtained another series of compounds derived from pyrrolo[3,4-*c*]pyridines [30]. This time, they studied the influence of other amino- and alkyl-(aryl-)aminoalkyl(hydroxyalkyl) substituents on the activity of 3,4-pyridinedicarboximide. They synthesized fifteen differently substituted derivatives, of which only compound **27** (Figure 22) deserved attention. Only compound 27 significantly depressed locomotor activity at doses of 1/10 and 1/20 LD_min_. [30].

The strong pharmacological activity of compound **27** encouraged scientists to introduce further modifications. They obtained a series of compounds in which the piperidine group present at position 2 was replaced with pyrrolidine or morpholine. Phenylpiperazine or pyrimidinylpiperazine was retained as an amine residue of the pharmacophore. The link between the core and the pharmacophore fragment was a propyl, butyl, or 2-hydroxypropyl chain [31].

**Figure 23:**
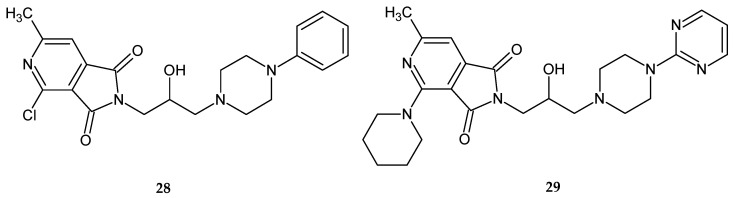
Pyrrolo[3,4-*c*]pyridines **28** and **29** significantly inhibited the spontaneous locomotor activity in mice.

This time, all the tested substances significantly inhibited the spontaneous locomotor activity of mice during a one-hour observation. Compounds **28** and **29** showed the most potent sedative effects (Figure 23). These compounds were active up to doses of 1/160 LD_50_ (**28**) and 1/80 LD_50_ (**29**).

The results indicate that replacing the piperidine ring at position 2 of the pyrrolo[3,4-*c]*pyridine scaffold with other pharmacophore substituents does not substantially alter the pharmacological action profile [31].

**Figure 24:**
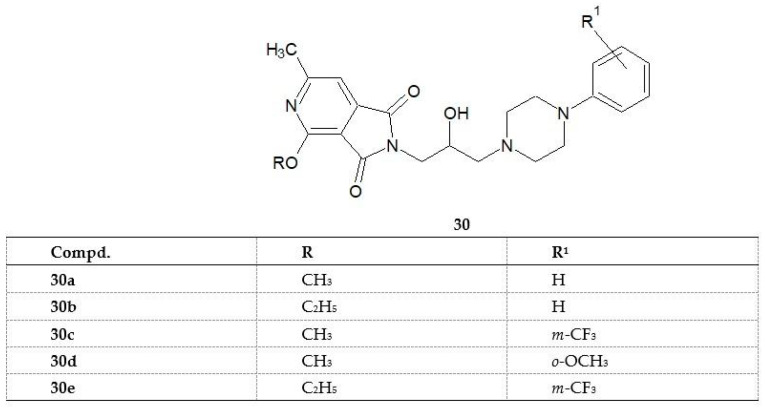
New derivatives of 2,3-dihydro-4-methoxy(ethoxy)-6-methyl-1,3-dioxo-1*H*-pyrrolo[3,4-*c*]pyridine **30a**–**e** [32].

Continuing the research, the scientists synthesized the next series of compounds in which the piperidine group was replaced by an alkoxy group [32]. The structures of compounds **30**–**34** are shown in Figure 24.

Compounds **30c**, **30d**, and **30e** significantly suppressed the spontaneous locomotor activity of mice during a 30 min observation up to a dose of 12.5mg/kg. Imide 31 was active at a dose of 200 mg/kg. All of the studied compounds (**30a**–**e**) exhibited analgesic activity [32]. It is described in the following section.

**Figure 25:**
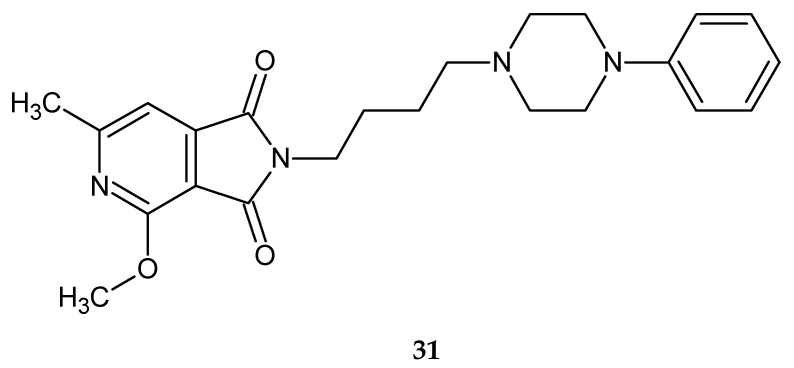
2-[(4-phenyl-1-piperazinyl)butyl]-4-methoxy-6-methyl-1*H*-pyrrolo[3,4-*c*]pyridine-1,3(2*H*)-dione **31**.

In the next publication [33], Śladowska’s team described the synthesis and properties of 2-(4-substituted)butyl derivatives of some 2,3-dihydro-1,3-dioxo-1*H*-pyrrolo[3,4-*c*]pyridines. New derivatives have an alkoxy group at position 2, butyl linker, and an amine residue (arylpiperazines, or 1,2,3,4-tetrahydroisoquinoline substituent) instead of a piperazinyl group. All tested compounds significantly suppressed the spontaneous locomotor activity of mice during a 30-min observation. While 2-[(4-phenyl-1-piperazinyl)butyl]-4-methoxy-6-methyl-1*H*-pyrrolo[3,4-*c*]pyridine-1,3(2*H*)-dione 31 (Figure 25), given in doses of 1/20, 1/40 and 1/80 LD_50_, inhibited spontaneous locomotor activity in mice by 86 (*p* < 0.02), 67 (*p* < 0.05), and 47%, respectively.

The other compounds in this series significantly reduced locomotor activity in mice, by 87–82% when administered at 1/20 LD_50_. However, when administered at doses of 1/40 and 1/80LD_50_, they decreased spontaneous locomotor activity by 59–47% and 26–13%, respectively [33].

In 2009, researchers reported [34] the synthesis and pharmacological results of novel 1*H*-pyrrolo[3,4-*c*]pyridine-1,3(2*H*)-diones. These compounds were obtained by modifying previously described structures [32,35,36]. The modifications consisted of the following:

- replacement of the phenyl ring at the N-4 position of piperazine with benzyl or benzhydryl groups;
- replacement of N-substituted piperazines by other cyclic amines (morpholine, piperidine, or pyrrolidine).

The authors aimed was to investigate if and how the performed structural changes would affect the toxicity, analgesic, and sedative effects of the tested compounds [34]. The results indicated that all tested pyrrolo[3,4-*c*]pyridine-1,3(2*H*)-diones significantly suppressed the spontaneous locomotor activity of mice during a 30-min observation.

**Figure 26:**
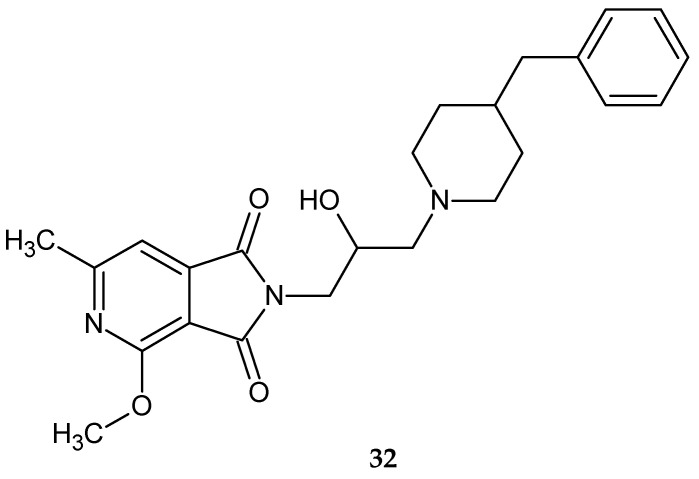
2-[2-hydroxy-3-(4-benzyl-1-piperidinyl)propyl]-4,6-dimethyl-1*H*-pyrrolo[3,4-*c*]pyridine-1,3(2*H*)-dione **32**.

The most potent effect (ED_50_ = 12.03 mg/kg) was produced by compound **32** (Figure 26). Moreover, compound **32** was not toxic (LD_50_ >2000 mg/kg) [34]. The antinociceptive activity of the pyrrolo[3,4-*c*]pyridines derivatives described in this publication is reported in the following section.

**Figure 27:**
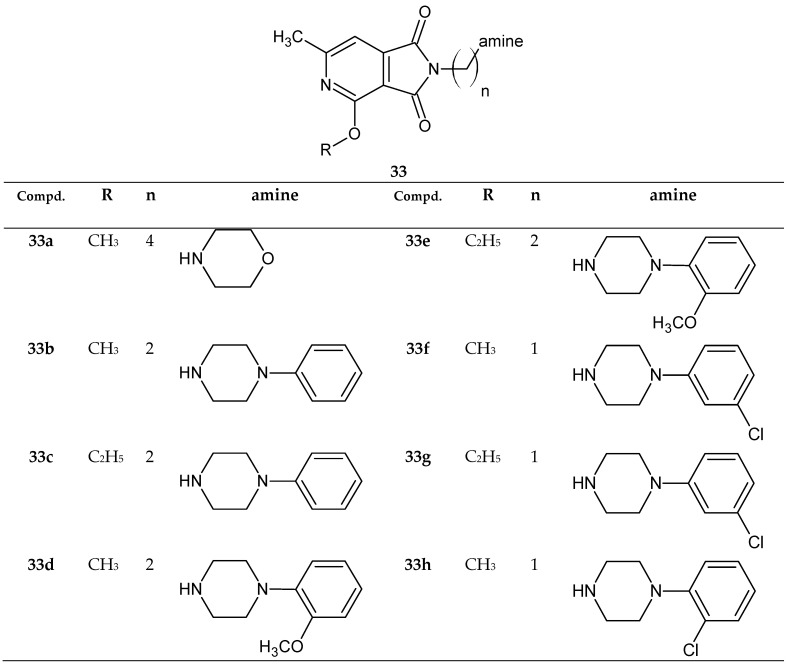
New derivatives of 1*H*-pyrrolo[3,4-*c*]pyridine-1,3(2*H*)-dione **33a**–**33h** with a sedative activity (and an analgesic activity).

In 2020, Szkatuła et al. [37] published a paper reporting the synthesis and pharmacological results of novel 1*H*-pyrrolo[3,4-*c*]pyridine-1,3(2*H*)-dione derivatives with potential sedative and analgesic activity. The structures of new derivatives **33a**–**33h** are shown in Figure 27 [37].

All new compounds **33a**–**h** inhibited the locomotor activity in mice, and the two most active (**33b**, **33d**) also extended the duration of thiopental anesthesia. Biological studies concerning imides **33b** and **33d** were supplemented with the determination of the effect of intraperitoneal administration of tested compounds on the duration of thiopental-induced sleep. The mechanism and extent of blood-brain barrier crossing were not defined. However, the observed sedative effect may indicate good penetration of the compounds in question into the central nervous system, which has not been proven either [37]. As mentioned earlier, the authors also described the analgesic activity of the new derivatives. Their results are presented in the section Analgesic activity.

### 2.6. Analgesic Activity of Pyrrolo[3,4-c]pyridine Derivatives

As mentioned above, Śladowska’s team initiated work on pyrrolo[3,4-*c*]pyridine derivatives to synthesize buspirone-inspired compounds. As a result of pharmacological studies, these compounds were found not to have the intended anxiolytic effect but to exhibit other pharmacological effects. The first structure that inspired the authors to work on analgesic pyrrolo[3,4-*c*]pyridine derivatives was the non-toxic compound **27** [27,30,31]. The researchers decided to modify the structure of this compound to obtain new pyrrolo[3,4-*c*]pyridine derivatives with potential analgesic activity. These modifications consisted mainly of replacing the pyridine present in the structure of **27** with another pharmacophore group. At position 2, the pyridine ring was replaced by morpholine, pyrrolidine, 4-methylpiperazine, or a chlorine atom. The analgesic effect of compounds was determined in two behaviorally different tests: phenylbenzoquinone-induced writhing syndrome test and hot-plate (thermal analgesic stimulus) test in mice. Unfortunately, most of the imides tested did not show any analgesic effect during testing [31].

Continuing their study, researchers observed that replacement of the piperidine group by an alkoxy one gave non-toxic substances with powerful analgesic properties [32]. The structures of compounds **30a**–**30e** are presented in Figure 24.

The analgesic action of the new 1*H*-pyrrolo[3,4-*c*]pyridine-1,3(2*H*)-dione derivatives **30a**–**30e** was investigated on mice using the hot-plate and writhing tests.

In both tests (writhing and hot-plate tests), all structures (**30a**–**30e**) tested showed analgesic activity superior to that of the reference acetylsalicylic acid (ASA). In the writhing test, imide **30a**, containing a methoxy group at position 2 of the pyridine ring and an unsubstituted phenyl at the N-4 position of piperazine, proved to be the most active compound. Replacement of the methoxy group with an ethoxy group (imide **30b**) contributed to an almost fourfold decrease in analgesic activity. Similarly, the activity of imide **30c** with a methoxy group in the pyridine ring was higher than that of its ethoxy analog **30e**. This result indicates that the potency of the analgesic effect in the writhing test is influenced by the type of alkoxy group at position 2 of the pyridine ring. The authors also observed that the introduction of trifluoromethyl or methoxy groups to the phenyl substituent caused a significant inhibition of spontaneous locomotor activity in mice. As it is known, analgesics can act centrally, peripherally, or both centrally and peripherally. Opioid analgesics, like morphine, base their analgesic effects mainly on a central mechanism, whereas non-steroidal anti-inflammatory drugs, like ASA, on a peripheral mechanism [38]. However, this division is not unambiguous as salicylates exert their analgesic effects also partly through the central mechanism [39]. Since the structures studied were active in both pharmacological tests, they may show analgesic effects both in the central and peripheral mechanism. However, as suggested by the authors, elucidation of the exact mechanism requires further pharmacological studies [32].

Continuing the research in 2005, Śladowska’s team designed another series of compounds with potential analgesic activity [35]. To this end, the researchers further modified the structure of 1*H*-pyrrolo[3,4-*c*]pyridine-1,3(2*H*)-diones **30a** and **30b** by:

- elimination of the hydroxyl group from the alkyl chain;
- introduction of a methoxy group at position 2 of the phenyl ring on N-4-piperazine;
- replacement of the phenyl ring with a 2-pyrimidine ring;
- replacement of the N-aryl(heteroaryl) piperazine group with tetrahydroisoquinoline and morpholine substituents;
- shortening of the alkyl chain at the nitrogen atom to C-1.

**Figure 28:**
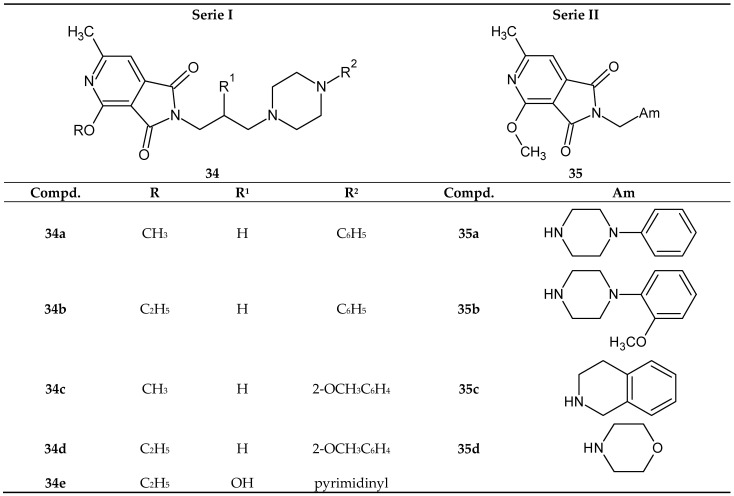
Derivatives of 1*H*-pyrrolo[3,4-*c*]pyridine-1,3(2*H*)-diones **34** and **35**.

Considering the above rationale, they synthesized the corresponding 1*H*-pyrrolo[3,4-*c*]pyridine-1,3(2*H*)-diones (Figure 28) to obtain compounds with potent analgesic activity [35].

The analgesic activity of compounds 34 and 35 was tested in two assays: hot-plate and writhing tests. In the writhing syndrome test, all tested substances, i.e., **34** and **35**, showed strong analgesic activity. The strongest effect was produced by compounds 34a and **34c**, which were effective up to a dose of 0.78 mg/kg. The most active compound in this test was imide **34c**, containing an o-methoxyphenyl substituent at the N-4 position of piperazine and a methoxy group at position 2 of the pyridine ring. None of the structures obtained was more active than the leading structure 30a in this test. This indicates that the most preferable modification involves removing a hydroxyl group at the β propyl position and introducing of a methoxy group at the ortho position of the phenyl ring. Only compound **35a** (the most toxic imide) was more active in the hot-plate test than compound **30a**. Compound **35a** showed significant activity up to a dose of 9 mg/kg in this test. In the case of 4-ethoxy derivatives, no increase in analgesic activities was observed concerning imide **30b**. The pharmacological results obtained indicate that most of the structures studied show very interesting analgesic properties. The results of preliminary radioligand binding studies suggest that these compounds show a weak affinity for µ-opioid receptors, which probably play a role in their mechanism of action. However, the authors do not explain the mechanism of action of the obtained pyrrolo[3,4-*c*]pyridine derivatives [35].

In 2006, the researchers obtained another series of compounds. This time, modifications to model structure **30a** involved introducing chlorine and fluorine atoms to the phenyl ring at the N-4 position of piperazine. Additionally, in several cases, the OH group was removed from the linker [36]. The analgesic activity of the compounds obtained in this study was measured using the writhing syndrome and hot-plate tests. All tested imides showed significant antinociceptive activity in phenylbenzoquinone-induced writhing test, and the ED_50_ values ranged from 3.51 to 16.04 mg/kg [36].

Importantly, these compounds were non-toxic. However, the analgesic activity (tested in both the hot-plate and writhing syndrome tests) of the compounds in this series was lower than the model structure **30a** previously adopted by the authors [34].

**Figure 29:**
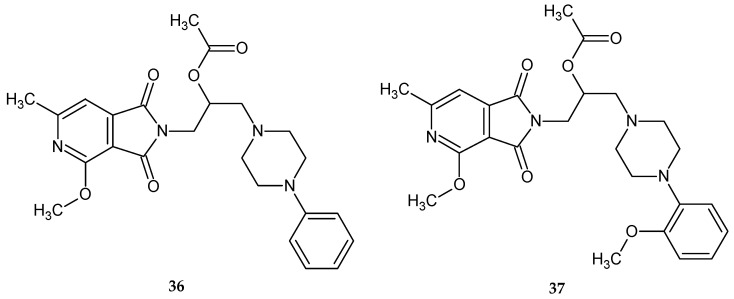
Derivatives of 1*H*-pyrrolo[3,4-*c*]pyridine-1,3(2*H*)-diones **36** and **37**.

In 2020, Dziubina et al. [40] published a paper that aimed to investigate the potential analgesic, antiedematous (anti-inflammatory) and antiallodynic activities using two 1*H*-pyrrolo[3,4-*c*]pyridine-1,3(2*H*)-dione derivatives **36** and **37** (Figure 29) in various experimental models of pain.

The authors performed a number of pharmacological tests, including the hot-plate test, formalin test, capsaicin test, and oxaliplatin-induced allodynia test. The hot-plate test is commonly used to evaluate centrally acting analgesics, and nociceptive responses in this test are of supraspinal origin. In the experiment conducted, only **36** at a dose of 20 mg/kg significantly increased the latency time of the pain response. However, as the authors suggest, its effect seems to be due to sedation (compound **36** at the highest analgesic dose significantly decreased locomotor activity) rather than antinociceptive action [40].

The formalin test is used to identify tonic inflammatory pain. It assumes two phases of stimulus perception – the first, lasting about 5 min after administration of an irritant (formalin), manifested by licking, shaking, biting of the paw by the animal, and the second, following several minutes, by tonic pain. The first phase results from the conduction of the pain stimulus along the C-fiber, whereas the second phase is the effect of increasing inflammation and central sensitization in response to a peripheral stimulus. In this test compounds **36** and **37** (5–20 mg/kg) showed antinociceptive activity in both phases, but it was more pronounced in the second phase of the test. Formalin-induced pain involves numerous channels, receptors, and signaling pathways; therefore, the authors verified the involvement of opioidergic, adenosinergic and nitrergic systems in the analgesic effects of compounds **36** and **37** in the model of tonic pain. The antinociceptive effect of both compounds was not reversed by systemic injection of naloxone, suggesting an opioid-independent analgesic effect of the test substances. Next, the effects of the compounds on the adenosinergic system were tested. In this test, caffeine (10 mg/kg), which by itself has no effect, reversed the effect of compound **37** on formalin-induced pain responses in both phases. Moreover, the antinociceptive effects of **37** were blocked by DPCPX (1,3-dipropyl-8-cyclopentyl-xanthine), an adenosine A1 antagonist, confirming that they were mediated by adenosine A1 receptors. As for the effects of these compounds on the nitrergic system (nitric oxide is involved in the analgesic effect of many drugs with different analgesia mechanisms), L-NAME (nω-nitro-L-arginine methyl ester hydrochloride) administration reduced the antinociceptive effect of compound **36** only in behaviors associated with the second phase. L-NAME administration did not affect the antinociceptive effect of compound **55**. The possible analgesic effect of compounds **36** and **37** on neurogenic pain was investigated using a capsaicin-induced pain model in mice. Capsaicin induces an immediate response at the application site, called neurogenic inflammation, which results from the activation of transient receptor potential vanilloid-1 and the release of mediators substance P and glutamate. Both compounds **36** and **37** reduced capsaicin-induced pain behaviors in a dose-dependent manner. In the next step of the study, the authors tested the potential anti-allodynic efficacy of the tested compounds **36** and **37** in a mouse model of chemotherapy-induced peripheral neuropathy (CIPN) pain induced by oxaliplatin, an anticancer drug commonly used in the model of neuropathy in humans. A single administration of oxaliplatin in animals (mice and rats) induces a painful peripheral neuropathy with associated mechanical allodynia. It is characterized by two phases: an early phase that develops soon after cytostatic administration (several hours after administration), and a late phase that occurs after several days [41]. The results of compounds **36** and **37** showed that as little as one dose of oxaliplatin decreased the threshold of pain sensitivity to mechanical stimuli, and tactile allodynia was observed as early as three hours after oxaliplatin injection. The observed effect of oxaliplatin was durable, as it was also found seven days after its administration. Test compounds **36** and **37** significantly reduced mechanical hypersensitivity at doses of 5 and 10 mg/kg. In the late phase of allodynia, both compounds **36** and **37** were significantly but slightly less effective than in the early phase of the test. The researchers also conducted in vitro studies. In this study, a biochemical assay was performed to determine the effect of the tested compounds on COX-2 levels. The COX-2 enzyme plays an essential role in the inflammatory response and its expression is induced after exposure of macrophages to LPS (lipopolysaccharide) or other pro-inflammatory stimuli. The RAW 264.7 cell line was used for this study. Compounds **36** and **37** significantly reduced COX-2 levels in LPS-stimulated cells. This result suggests at least partial anti-inflammatory properties of the tested compounds.

The obtained pharmacological results showed that compounds **36** and **37** exhibits broad-spectrum activity in several pain models (neurogenic pain, inflammatory pain, and chemotherapy-induced peripheral neuropathic pain) [40].

**Figure 30:**
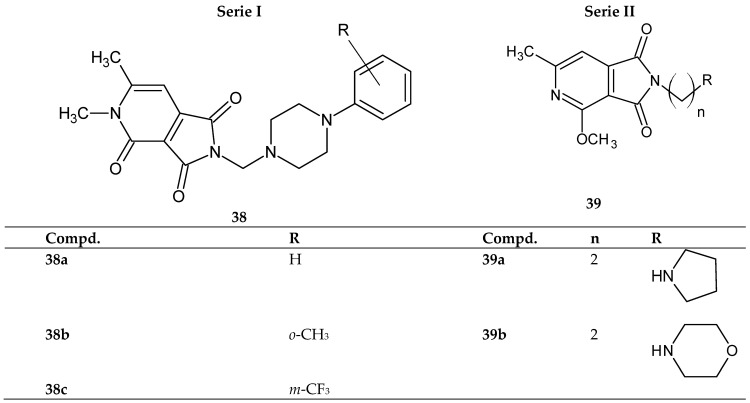
Derivatives of 1*H*-pyrrolo[3,4-*c*]pyridine-1,3(2*H*)-diones **38** and **39**.

Krzyżak et al. [42] synthesized a novel N-substituted 1*H*-pyrrolo[3,4-*c*]pyridine-1,3(2*H*)-diones derivatives **38**–**39**. The compounds were tested for their inhibitory activity against COX-1/ COX-2 and BSA (bovine serum albumin) interaction. The researchers obtained two series of compounds (Figure 30). Series I—Mannich bases with an arylpiperazine moiety **38a**–**38c**. Series II—structures with a two-carbon linker and a cyclic amine **39a**, **39b** (Figure 30) [42].

In vitro, COX-1 and COX-2 inhibition assays were performed, and pharmacological results showed that all compounds **38**–**39** have the potential to inhibit both enzymes. It should be noted that COX-1 is involved in the synthesis of prostaglandins responsible for maintaining normal body function in the kidneys, intestines, and other organs, while COX-2 is an isoform that plays a major role in inflammation and associated pain [43,44]. In the study presented by the researchers, the COX-1 enzyme is inhibited more effectively (than the reference meloxicam) by compounds **38a** and **39a**, while COX-2 is inhibited more strongly than the reference drug by all compounds (except **38c**). The highest selectivity towards the COX-2 enzyme was shown by structure **39a**, for which the COX selectivity ratio (IC_50_(COX-2)/IC_50_(COX-1)) is 0.55 (for meloxicam—0.71) [42].

The interaction with BSA was investigated by fluorescence spectroscopy and circular dichroism measurement. To understand the binding interaction of compounds **38**–**39** in the active site of COX and BSA, a molecular docking study was performed [42].

The obtained experimental and molecular docking results confirmed that the main interaction forces between the studied **38**–**39** structures and BSA are hydrogen bonding and van der Waals forces [42].

As mentioned in the previous section, compounds **33a**–**33h** (Figure 27) presented interesting antinociceptive activity in addition to the strong sedative activity that has already been described [37]. Compounds 33a–33h (except **33c**) were not toxic (LD_50_ >2000mg/kg). The analgesic activity of new compounds was studied using the hot-plate test and the writhing test. All tested 3,4-pyridinedicarboximide **33a**–**33h** were active in the writhing test (ED_50_ = 3.25–19.2 mg/kg), and their analgesic activities in this study exceeded the effect of the reference ASA (ED_50_ = 39.15 mg/kg). In addition, the pharmacological activity of the two imides **33b** (ED_50_ = 3.25 mg/kg and **33d** (ED_50_ = 3.67mg/kg) was comparable to that of morphine used as a second reference drug (ED_50_ = 2.44 mg/kg). However, in the case of the hot-plate test, the analgesic effects observed for the tested structures did not reach statistical significance [37].

**Figure 31:**
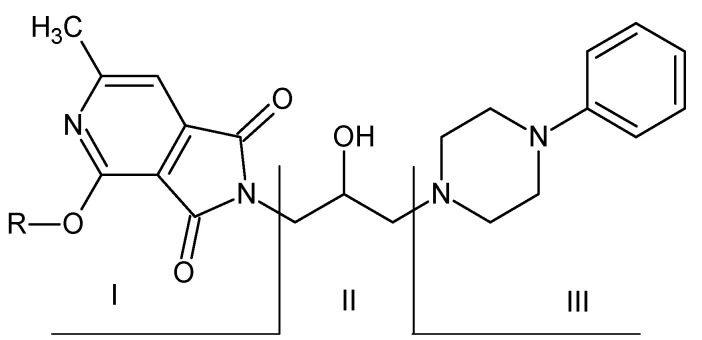
Structure of imides. (I) 3,4-pyridinedicarboximide (R=CH_3_, C_2_H_5_), (II) linker, (III) type of amino residue [37].

Based on the obtained results and taking into account the results obtained in previous studies [31,33,34,35], the authors aimed to determine the relationship between the structure and biological activities in the group of N-substituted 1*H*-pyrrolo[3,4-*c*]pyridine-1,3(2*H*)-diones. In their results, the researchers considered the effect of the following modifications on pharmacological activity (Figure 31):

(I) an alkoxy substituent in the pyridine ring;
(II) the length of the alkyl link;
(III) the type of amino residue.

The potency of the analgesic effect of the studied compounds is decisively influenced by the type of alkoxy substituent in the pyridine ring (I). Compounds substituted with methoxy group in this position are more active than their ethoxy analogues.

In most cases, a phenylpiperazine moiety was introduced as the amine residue (III). The most active compounds had an unsubstituted phenyl ring in this grouping. Introduction of alkyl substituents at the ortho or meta position (*o*-OCH_3_, *m*-CF_3_) or halogen atoms was not preferred. Replacement of the phenylpiperazine moieties with another cyclic amine (morpholine, tetrahydroisoquinoline) also led to attenuation of the analgesic activity. The length of the alkyl bond between the pyrrole ring (II) and the amine residue (III) also affects the analgesic potency. Removal of the hydroxyl group weakens the pharmacological effect. Shortening of this linker to 2 or 1 carbon atom also causes a decrease in activity, whereas lengthening the linker to 4 carbon atoms (butyl derivatives) did not increase the potency [37].

## 3. Conclusions

Pyrrolo[3,4-*c*]pyridines are biheterocyclic compounds containing a pyrrole ring condensed with a pyridine ring in their structure. The pharmacophore groups, which determine the biological properties of pyrrolopyridines, are most often attached to the pyrrole ring. The review of collected literature shows that carbonyl substituents in the pyrrole ring significantly impact the activity of pyrrolopyridines. The imide structure of such structures determines their physicochemical properties. Imide-based compounds are often neutral and hydrophobic, which determines their ability to penetrate biological membranes [45,46]. Pyrrolo[3,4-*c*]pyridine derivatives have not been introduced into medicine so far, however, as it results from the studies presented in the literature, they exhibit various pharmacological activities. Derivatives with analgesic and sedative properties are by far the largest group [27,28,29,30,31,32,33,34,35,36,37,40,42]. Their antidiabetic [8,9,10], antiviral [12,13,14,15], antimycobacterial [16,17,18,19], and anticancer [20,21,22,23,24] activities have also been described. They can also be used in diagnostics as chemosensors to image Fe^3+^ in living HepG2 cells [47]. The diverse profile of biological activity may be an incentive to continue research into this group of compounds.
